# Outsourcing the Management of Reusable Medical Devices in a Chain-Wide Care Setting: Mixed Methods Feasibility Study

**DOI:** 10.2196/41409

**Published:** 2023-09-19

**Authors:** Bart A C Noort, Paul Buijs, Oskar Roemeling

**Affiliations:** 1 Department of Operations Faculty of Economics and Business University of Groningen Groningen Netherlands; 2 Department of Innovation Management & Strategy Faculty of Economics and Business University of Groningen Groningen Netherlands

**Keywords:** health care logistics, outsourcing, web ordering portal, medical devices, feasibility study, device management

## Abstract

**Background:**

Managing reusable medical devices incurs substantial health care costs and complexity, particularly in integrated care settings. This complexity hampers care quality, safety, and costs. Studying logistical innovations within integrated care can provide insights to medical devices use among staff effectively.

**Objective:**

This study aimed to establish the feasibility of a logistical intervention through outsourcing and a web portal. The goal was to provide insights into users’ acceptability of the intervention, on whether the intervention was successfully implemented, and on the intervention’s preliminary efficacy, thus benefiting practitioners and researchers.

**Methods:**

This paper presents a mixed methods feasibility study at a large chain-wide health care provider in the Netherlands. The intervention entailed outsourcing noncritical reusable medical devices and introducing a web portal for device management. A questionnaire gauged perceived ordering and delivery times, satisfaction with the ordering and delivery process, compliance with safety and hygiene certification, and effects on the care delivery process. Qualitative data in the form of observations, documentation, and interviews were used to identify implementing challenges. Using on-site stocktaking and data from information systems, we analyzed the utilization, costs, and rental time of medical devices before and after the intervention for wheelchairs and anti–pressure ulcer mattresses.

**Results:**

Looking at the acceptability of the intervention, a high user satisfaction with the ordering and delivery process was reported (rated on a 5-point Likert scale). With respect to preliminary efficacy, we noted a reduction in the utilization of wheelchairs (on average, 1106, SD 106 fewer utilization d/mo), and a halted increase in the utilization of anti–pressure ulcer mattresses. In addition, nurses who used the web portal reported shorter ordering times for wheelchairs (−2.7 min) and anti–pressure ulcer mattresses (−3.1 min), as well as shorter delivery times for wheelchairs (−0.5 d). Moreover, an increase in device certification was reported (average score of 1.9, SD 1.0), indicating higher levels of safety and hygiene standards. In theory, these improvements should translate into better outcomes in terms of costs and the quality of care. However, we were unable to establish a reduction in total care costs or a reduced rental time per device. Furthermore, respondents did not identify improvements in safety or the quality of care. Although implementation challenges related to the diverse supply base and complexities with different care financers were observed, the overall implementation of the intervention was considered successful.

**Conclusions:**

This study confirms the feasibility of our intervention, in terms of acceptability, implementation success, and preliminary efficacy. The integrated management of medical devices should enable a reduction in costs, required devices, and material waste, as well as higher quality care. However, several challenges remain related to the implementation of such interventions.

## Introduction

### Background

Managing medical devices is a complex but important element in the delivery of high-quality care and has considerable effect on the financial bottom line of health care providers [1,2]. If not managed well, there can be various downsides, such as health care organizations holding too many devices in relation to demand [3,4], physicians needing to wait for delivery of the devices they need urgently [5], nurses possibly not having enough time available for the direct care process [6], and using devices that do not comply with safety and hygiene standards [7]. Hence, health care organizations spend considerable time and resources on seeking solutions to mitigate these problems, such as opting for joint procurement, applying lean manufacturing principles, using tracking technologies, and outsourcing [2,8]. In this paper, we are especially interested in outsourcing as a potential route to better medical device management.

Achieving better management of medical devices is particularly challenging when one considers that health care providers are still working toward more integrated care delivery [9]. Although such integrated care developments seem promising, operational and organizational challenges are often encountered when operating across care disciplines [10,11]; for example, patients may appreciate being able to retain the same wheelchair when moving between care locations, but this creates additional logistics or administrative tasks for nursing staff. Furthermore, the stringent requirements on the traceability and safety of medical devices under the 2021 European Union (EU) Medical Device Regulation challenge manufacturers and care providers to improve their medical device management [12]. The outsourcing of logistics activities may address some of these challenges by reducing coordination and planning efforts and improving their results [13,14]. Whether such outsourcing solutions, in this case related to managing medical devices, would be perceived as acceptable by nursing staff and indeed improve care delivery need to be carefully assessed.

When pursuing integrated care delivery, supportive IT becomes pivotal, including in relation to medical device management [15]. Care professionals generally have a positive attitude toward the implementation of IT [16]. However, its implementation and adoption are complicated by the multistakeholder context involving patients, medical staff, and other organizational staff [17]. Previous studies have underlined the importance of the usability of technology in gaining the support of core medical staff, thereby contributing to a successful implementation and increasing the likelihood of a positive effect on care delivery and other outcomes [18,19]. In this study, we focused on the feasibility of using a web portal aimed at enhancing medical device management through outsourcing.

### Research Aims

We conducted a mixed methods study to assess the feasibility of a logistical intervention, where a health care provider outsourced several noncritical reusable medical devices to a third-party provider and introduced a web portal to support medical device management. Feasibility is based on the extent to which an intervention is considered acceptable by the users; whether the intervention is successfully implemented; and, finally, the intervention’s preliminary efficacy. Our aims with regard to outsourcing were to reduce the (1) total utilization days of the medical devices; (2) delivery time; and (3) time and money spent on cleaning, maintaining, and internally transporting the devices. We aimed to achieve our goals by using a web portal that supports medical staff by reducing the time taken to order a device when needed and to deregister it when no longer needed and that provides them with easy insight into device availability from their own location. With this study, we provide practitioners and researchers with knowledge on the potential benefits of, and challenges related to, outsourcing medical devices, assisted by IT, in an integrated care provider setting.

## Methods

### Research Context

The study took place between January 2019 and June 2022 in the context of a large regional health care provider in the Netherlands. The organization, which was established after a merger of a hospital, several nursing homes, and home care providers, consists of 1 hospital, 17 nursing homes, and 3 residential care centers; it also provides home-based care at several locations. Since the merger, the health care provider has moved toward becoming a more integrated care organization (eg, by implementing an organization-wide electronic medical record system). It was the first such organization to receive chain-wide accreditation worldwide from the Joint Commission International [20].

The Dutch health care system is organized around 3 domains, largely based on the principles of *managed competition* [21]. The curative care domain is regulated by the Health Insurance Act. Private health insurers compete for beneficiaries, whereas private not-for-profit health care providers compete for patients. The long-term care domain is regulated under the Dutch Long-term Care Act, with a national tax-based budget. Budgeting responsibility for long-term care is regionally executed by so-called care offices. The home care domain falls under the responsibility of local government, regulated by the Social Support Act. Given that the studied health care provider is active in all 3 care domains—hospital, residential, and home care—it has contractual agreements with all regional financing bodies: health insurers, care offices, and local government. This leads to a complex setting where the health care provider is required to determine which medical device is associated with which financing body and to understand the conditions that govern the use of these specific devices. Within this health care context, we focused on the management of noncritical reusable medical devices. Reusable medical devices are defined as those that health care providers can reprocess and reuse for >1 patient [22]. Typically, reusable medical devices require calibration, maintenance, repair, user training, cleaning, and decommissioning [23]. In our research, noncritical devices are typified by the fact that they are not invasive, such as wheelchairs and patient lifts. Critical or semicritical medical devices, such as surgical instruments or equipment for diagnostics [22], are beyond the scope of our research.

Before the intervention, on-site stocktaking showed that several medical devices were unused, and there was little insight into the actual stock held. Approximately 40% of the devices physically identified were not registered, whereas several devices registered as available on-site were untraceable. Exploratory meetings with staff and on-site observations indicated that the existing way of managing devices led to considerable time spent searching by employees, patients having to wait, and high inventory and management costs.

Our focus on reusable medical devices provides a setting demarcated by relatively low complexity in which to study how to structurally benefit from logistical solutions such as outsourcing and web ordering portals in a chain-wide provider.

### Intervention

The intervention consisted of 2 parts. The first part involved a transition to the full outsourcing of 8 types of medical devices: anti–pressure ulcer mattresses, bariatric care beds, bed trapezes, lifting slings, low-low beds, patient lifts, standard wheelchairs, and shower stretchers. Of these, bed trapezes, lifting slings, low-low beds, shower stretchers, and standard wheelchairs were largely (>90%) managed and owned by the health care provider before the intervention. Anti–pressure ulcer mattresses, bariatric care beds, and patient lifts were already outsourced for most care locations (>90% of the devices were outsourced). From April 2021 onward, all 8 device types have been fully outsourced. Since then, the health care provider has been paying a rental fee per device per day to a third-party medical device supplier that transports, stores, cleans, and maintains the devices. The second part of the intervention involved implementing a web portal for nurses to support and simplify the process of registering and deregistering an increased number of rented medical devices (implemented on September 1, 2021). Before the intervention, nurses ordered medical devices via telephone or email, either via the internal purchasing department or directly through an external device supplier. The portal provides a standardized system for ordering these devices and gives nursing staff insight into the number of devices ordered and the devices currently available on-site. Overall, the intervention aimed to unify the management of noncritical reusable medical devices across the entire organization.

### Study Setup

This study can be typified as a mixed methods feasibility study. It is based on several data sources: medical device rental data, interviews, on-site observations, and a small-sample questionnaire. The study has a before-and-after design and addresses 3 of the typical goals of feasibility studies, as classified by Bowen et al [24]: whether the outsourcing of reusable medical devices and the introduction of a web ordering portal are acceptable, implementable, and effective.

**Figure 1:**
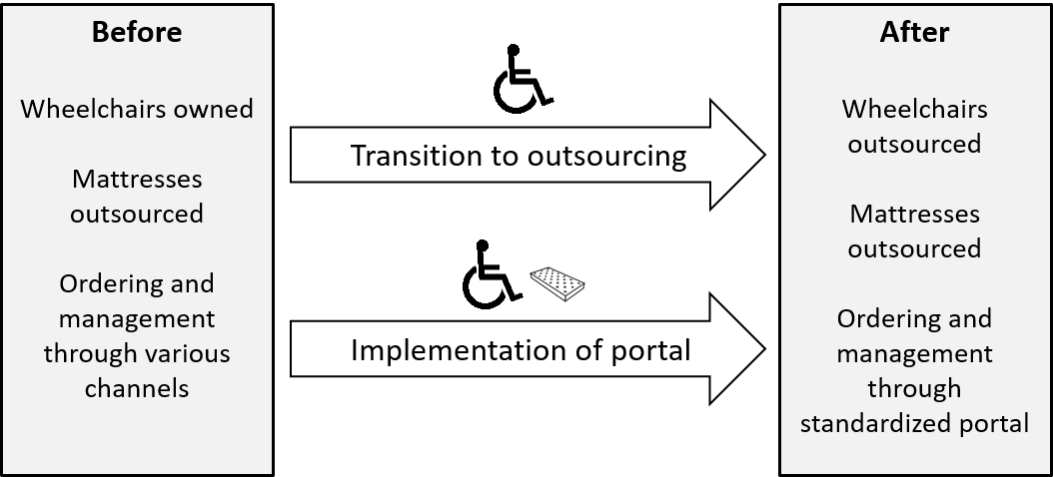
Schematic overview of the intervention: transition from owning to outsourcing wheelchairs and the implementation of a web portal for ordering wheelchairs and anti–pressure ulcer mattresses.

In this paper, we focused on 2 types of devices: wheelchairs and anti–pressure ulcer mattresses. Each device type was exposed to different aspects of the intervention during our study: wheelchairs were undergoing the transition to outsourcing and the implementation of the web portal, whereas, for anti–pressure ulcer mattresses, the web portal was the main change in managing the devices. A comparison between these 2 types of devices thus enables a feasibility assessment of the transition to outsourcing separately from the implementation of the web portal. A schematic overview of the intervention is shown in Figure 1.

### Web Portal Data Collection

#### Stocktaking and Rental Data

To establish the utilization days of the devices managed and owned by the health care provider before the intervention, device stocktaking was undertaken at all care locations in 2019. The aim was to establish (1) whether devices that were shown in the enterprise resource planning (ERP) system were present on-site and whether devices on-site were shown in the ERP system, (2) whether devices had received maintenance within the required period, and (3) whether devices were ready for use in terms of cleanliness and functioning. The utilization days of outsourced devices were determined based on rental data from the third-party supplier that showed the number of newly ordered devices and the rental time per device for each care location. Medical devices used for providing home care are not included in our analysis because, under the Dutch Long-term Care Act, they are not the responsibility of the care provider.

#### Interviews and On-Site Observations

We collected qualitative data through on-site observations and interviews to understand the supply and management of noncritical reusable medical devices; to explore areas for improving delivery time, patient care, cost reduction, and nursing staff satisfaction; and to reveal potential challenges that may occur when implementing these changes. Available supporting documentation was also analyzed. This data collection also helped explain how the transition to outsourcing and the implementation of the web portal came into effect. During 2 rounds of interviews (2019 and 2021), a total of 30 interviews carried out in Dutch were held with internal and external stakeholders to provide deeper insight into challenges regarding the management of noncritical reusable medical devices. The interviewed participants were nursing staff, team leaders, care location managers, logistics managers, and care purchasers from municipalities and health insurers. The interview guide (translated into English) can be found in Multimedia Appendix 1.

#### User Experience and Care Quality Questionnaire

User satisfaction related to the intervention was evaluated by means of an anonymous questionnaire (in Dutch). After the intervention, between April 28, 2022, and June 8, 2022, after an informal announcement by the project leader of the care provider, nurses from all care locations were approached by email and invited to complete a questionnaire. Questions related to the ease of use of the web portal, ordering and delivery times for devices, and the perceived consequences of the intervention in terms of the quality of care and patient satisfaction. Each question had responses using a 5-point Likert scale ranging from 1=*fully agree* to 5=*fully disagree*. The 2 types of devices, wheelchairs and anti–pressure ulcer mattresses, were used as examples in the questionnaire. The questionnaire (translated into English) can be found in Multimedia Appendix 2.

An overview of the data collection and timing is provided in Table 1.

**Table 1:** An overview of the used data sources, linked to the different study phases, as well as study objectives.

Data source	Phase of data collection	Objective
Stocktaking	Before the intervention	Preliminary efficacy
Rental data	Before and after the intervention	Preliminary efficacy
Interviews and observations	Before and after the intervention	Acceptability Implementation success
Questionnaire	After the intervention	Acceptability Preliminary efficacy

### Data Analysis

The utilization days of owned (wheelchairs) and rented (anti–pressure ulcer mattresses) devices before the intervention were compared with the situation after the intervention using stacked charts, showing the utilization of medical devices in days per month per care location. The stocktaking data provided the number of owned and rented devices present at each care location before the intervention. All the various device brands and models were clustered into basic types (wheelchairs or anti–pressure ulcer mattresses) to ensure comparability before and after the intervention. The number of owned devices was converted to utilization in days per month. Average rental time per newly rented device was calculated and analyzed for 4 care locations (rental data only). Total device utilization and average utilization time were analyzed by means of 2 linear regression models (Excel 2016 [Microsoft Corporation]). Table 2 provides an overview of the dependent and independent variables, which were defined before the analysis. Autocorrelation between outsourcing and the portal was checked based on variance inflation factors and was <3 in all models.

The questionnaire data were analyzed by distinguishing three user groups: (1) nurses who used the web portal for both wheelchairs and anti–pressure ulcer mattresses, (2) nurses who used the web portal for only 1 type of device, and (3) nurses who had never used the web portal or had stopped using it. The questionnaire responses were analyzed by comparing the average difference between reported ordering time and delivery time before and after the intervention and on the average score per item on questions regarding ordering satisfaction and the perceived effects of the intervention. Differences in average ordering satisfaction and perceived effects among the user groups were assessed by means of a 2-tailed *t* test (Excel 2016). Differences in the average reported ordering time and delivery time before and after the intervention were assessed by means of a paired 2-tailed *t* test.

The interview data were analyzed with the aim to thoroughly understand the challenges that emerged from the transition to outsourcing and the implementation of the web portal—and how the care provider dealt with these challenges—as well as to highlight the challenges that the intervention did not resolve. Led by the first author, we performed the analysis using ATLAS.ti software (ATLAS.ti Scientific Software Development GmbH) and Excel, following the 3-step grounded theory approach of Corbin and Strauss [25] as discussed in the study by Karlsson [26]. This process follows a cycle where categories are inductively formed from the data, followed by a deductive analysis where findings are refined and validated based on existing concepts. In the first step, open coding, the transcripts were summarized and short text codes assigned. In the second step, axial coding, we established more general categories related to typical characteristics, challenges, enabling conditions, and consequences related to medical device use within the health care organization. In the third step, selective coding, 2 typical patterns emerged that seemed to have an effect on the implementation process of the portal and the outsourcing contract. The first pattern related to the scale of uptake of the intervention, leading to ambiguity in the organization. The second pattern concerned the effect of market incentives, hampering suppliers in offering a wide product portfolio. The coding process and intermediate outcomes were frequently discussed between the first and second authors to achieve consensus in categorization and established patterns.

**Table 2:** An overview of the linear regression models and included variables.

Model	Dependent variable	Independent variables
1a	Total device utilization (wheelchairs)	Time (mo), web portal (dummy), and outsourcing (dummy)
1b	Total device utilization (anti–pressure ulcer mattresses)	Time (mo) and web portal (dummy)
2a	Average device utilization time (wheelchairs)	Time (mo), web portal (dummy), outsourcing (dummy), and location
2b	Average device utilization time (anti–pressure ulcer mattresses)	Time (mo), web portal (dummy), and location

### Ethical Considerations

The data regarding rental of medical devices were routinely collected by the health care provider for administrative purposes and not linked to individual patients. The data processed in this study are not considered to constitute medical research involving human participants as defined in the 1964 Helsinki declaration. As such, the statutes of the institutional review board of the faculty of economics and business of the University of Groningen indicate that the study does not require ethics approval by a review committee. Nevertheless, all the methods described in this study were conducted in accordance with the 1964 Helsinki declaration and its later amendments or comparable ethical standards.

All interview and questionnaire respondents were informed about the purpose of the data collection and the data processing procedure, and they consented to participate.

## Results

### Overview

The results are presented in 3 parts. In the first part, we evaluated the acceptability of the intervention by nursing staff. In the second part, we addressed to what extent the intervention was successfully implemented and what enabling or limiting factors have been established. Finally, in the third part, we offered a preliminary assessment of the efficacy of the intervention in terms of device utilization, costs, ordering and delivery time, quality of care, and compliance with safety and hygiene standards.

### Acceptability

#### Questionnaire Responses

User satisfaction, as measured in the questionnaire responses, indicates how the nursing staff rate the new ordering process that is based on outsourcing and the web portal. Nurses (N=45) who were responsible for ordering reusable medical devices completed the questionnaire. Of the 45 nurses, 28 (62%) reported using the web portal for ordering both wheelchairs and anti–pressure ulcer mattresses, 10 (22%) used the web portal only for anti–pressure ulcer mattresses and not for wheelchairs, and 7 (16%) reported not using the web portal and sticking with the earlier procedure of telephoning and emailing.

#### Satisfaction With the Ordering Process

User satisfaction with the ordering process, with specific attention to the use of the web portal, was generally rated highly (Table 3). Nurses who ordered both types of devices through the web portal reported an average satisfaction score of 1.6 (SD 0.6; using a Likert scale ranging from 1 to 5, with 1=*most satisfied* and 5=*least satisfied*). Nearly half (24/45, 53%) of the respondents fully agreed with the statement that the ordering process is easy. Nurses who only ordered anti–pressure ulcer mattresses via the web portal reported an average satisfaction score of 2.3 (SD 0.6), which was a statistically significant (*P*=.003) lower rating than that of nurses who ordered both types of equipment via the web portal. Respondents who did not order both types of equipment via the web portal (17/45, 38%) reported either being unaware of the web portal, unsure of how to use it, or dissatisfied with its functionality.

**Table 3:** Satisfaction with the web portal ordering process (rated using a 5-point Likert scale ranging from 1=fully agree to 5=fully disagree)^a^.

	Ordering is easy, mean (SD)	Ordering is clear, mean (SD)	Ordering is fast, mean (SD)	The portal is accessible, mean (SD)	The portal is stable, mean (SD)	The portal is user friendly, mean (SD)	Score, mean (SD)
Ordering via the web portal (n=28)	1.7 (1.0)	1.8 (1.1)	1.6 (0.9)	1.6 (0.7)	1.5 (0.7)	1.7 (0.8)	1.6 (0.6)
Ordering mattress via the web portal and wheelchair via email or telephone (n=10)	1.8 (0.9)	1.9 (0.9)	1.9 (1.0)	1.7 (0.9)	1.8 (0.9)	2.0 (0.7)	2.3 (0.6)
Average	1.7 (0.9)	1.8 (1.1)	1.7 (0.9)	1.6 (0.8)	1.6 (0.8)	1.8 (0.8)	1.8 (0.7)

^a^Overall satisfaction with ordering via web portal versus satisfaction with ordering mattress via portal: *P*=.003 (average scores, based on a 2-tailed *t* test with unequal variances).

#### Satisfaction With Device Management

With respect to user satisfaction with medical device management in general, the *insight into availability* dimension was rated the highest, with an average rating of 1.8 (SD 1.1; Table 4). Of the 45 respondents, 27 (60%) fully agreed with the statement that there is now a better insight into available devices. Whether there was an improvement in the product range was rated with an average score of 2.1 (SD 1.0). Perceived increase in the ease of transferring devices between locations was rated the lowest (2.4, SD 1.3), which is a score suggesting only a minor improvement. No statistically significant differences between users or (partial) nonusers of the web portal were found.

**Table 4:** Overall satisfaction with the intervention (rated using a 5-point Likert scale ranging from 1=fully agree to 5=fully disagree)^a^.

		Product range is larger, mean (SD)	Better insight into available devices, mean (SD)	Easier to transfer devices between care locations, mean (SD)	Score, mean (SD)
Ordering via the web portal (n=28)		2.1 (1.0)	1.7 (1.1)	2.1 (1.3)	2.0 (0.9)
**Ordering mattress via the web portal and wheelchair via email or telephone (n=10)**					
	Wheelchair	2.1 (1.2)	1.7 (1.0)	2.3 (1.0)	2.0 (0.9)
	Mattress	2.0 (0.9)	1.3 (0.7)	3.0 (1.5)	2.1 (0.8)
Ordering via a different method (n=7)		2.1 (1.1)	2.4 (1.1)	2.0 (1.2)	2.2 (0.9)
Average		2.1 (1.0)	1.8 (1.1)	2.4 (1.3)	2.0 (0.9)

^a^Overall satisfaction with ordering via web portal versus satisfaction with ordering mattress via portal (wheelchair): *P*=.92, overall satisfaction with ordering via web portal versus satisfaction with ordering mattress via portal (mattress): *P*=.80, overall satisfaction with ordering via web portal versus satisfaction with ordering via different method: *P*=.63, overall satisfaction with ordering mattress via web portal (wheelchair) versus satisfaction with ordering mattress via web portal (mattress): *P*=.91, overall satisfaction with ordering mattress via web portal (wheelchair) versus satisfaction with ordering via different method: *P*=.74, and overall satisfaction with ordering mattress via web portal (mattress) versus satisfaction with ordering via different method: *P*=.81 (average scores, based on a 2-tailed *t* test with unequal variances).

### Implementation

#### Overview

The acceptability assessment in the previous subsection indicates generally positive findings, and the implementation of the intervention was successful for all 8 types of medical devices. Nevertheless, evaluating the implementation of the intervention also highlighted a number of challenges. The limited uptake of the web portal by nurses (31/45, 69%) shows that there is room for improvement. On the basis of the observational and interview data, we explain in the following subsections how the implementation of the intervention is hampered by the complex context of the care market in which this chain-wide care provider is embedded.

#### Uptake of the Improved Ordering Process

Although most of the nurses reported that they do follow the new ordering process, several mentioned that they still use other ordering methods, such as ordering via email or telephone or by ordering from other suppliers. To an extent, this is inevitable in the current situation because some of the more specialized devices, such as customized wheelchairs, are only available from other suppliers and not from the one involved in the intervention. A problem is that this then leads to ambiguity about what ordering process should be used for which type of device, thereby structurally hampering a full uptake of the intervention. This is exemplified by several remarks in response to the questionnaire:

I do not order wheelchairs via [supplier of study] but via the occupational therapy department, who arrange it with another supplier.

Head nurse 1

Besides working as a nurse, I also work as an occupational therapy coach, so I order devices via email.

Head nurse 1

In addition, nurses reported that there are still many device types where the internal ordering process is experienced as too cumbersome and slow:

I particularly have problems with the purchasing process when something is not available from [supplier of study]...I need to go through the purchasing and infection prevention department, the whole process sometimes takes 4 to 6 weeks. The patient may have already passed away by then.

Occupational health nurse 2

These quotations illustrate that nursing staff will only fully adapt to, and benefit from, the new ordering process when it is implemented on an organization-wide scale, with a larger range of device types available. However, given the diverse supply base as well as the various care financers involved, this seems challenging.

#### Effect of a Complex Market

At the outset of our study, we expected the chain-wide health care provider in this study to have a unique opportunity to simplify the management of reusable medical devices; namely, because the provider is active across the 3 care domains in the Netherlands (ie, curative care, long-term care, and social care), we expected that it would be able to pool devices across these domains, thereby reducing its device supply base and the number of ordering methods. However, in practice, device suppliers tend to specialize their service and product range to a specific care domain, thereby forming a barrier to fulfilling our initial expectations:

It is partly a very practical issue. Device suppliers have contracts with a selection of health care providers and device manufacturers...When it comes to maintenance, some spare parts can only be supplied by a specific manufacturer, which does not always align with the device type demanded by the health care provider. At the same time, our goal is—and it will be the same for [name of care provider]—to deliver affordable and personalized care for clients, and that sometimes creates tensions.

Policy advisor of municipality

Furthermore, the care path of a single patient can span multiple domains. In this situation, the ordering methods and device suppliers ordinarily change during the care process. Consequently, to meet individual patient needs and to prevent long waiting times, nursing staff put much effort and time into the process of ordering devices. Essentially, they take care of the service and coordination duties that should partly be the responsibility of device suppliers and care financers:

When a patient goes home after rehabilitation, we sometimes sell them our bed trapeze; otherwise, the patient has to order it, and it may not arrive in time. Also, we may order a wheelchair for short-term use under the Social Care Act from the supplier. If a patient needs longer-term care, we also order wheelchairs under the Long-term Care Act or other devices via the care insurer...So, unfortunately, we put quite some time and effort into coordinating the device process for patients.

Occupational health nurse 1

These findings show that the multitude of stakeholders and processes that are part of health care markets limit the extent to which nursing staff benefit from the transition to outsourcing and the implementation of the web portal and hamper a structural implementation of such solutions.

### Preliminary Efficacy

On the basis of comparing the use of wheelchairs and anti–pressure ulcer mattresses before and after the intervention, we now present the findings of our preliminary efficacy analysis.

#### Utilization of Reusable Medical Devices

**Figure 2:**
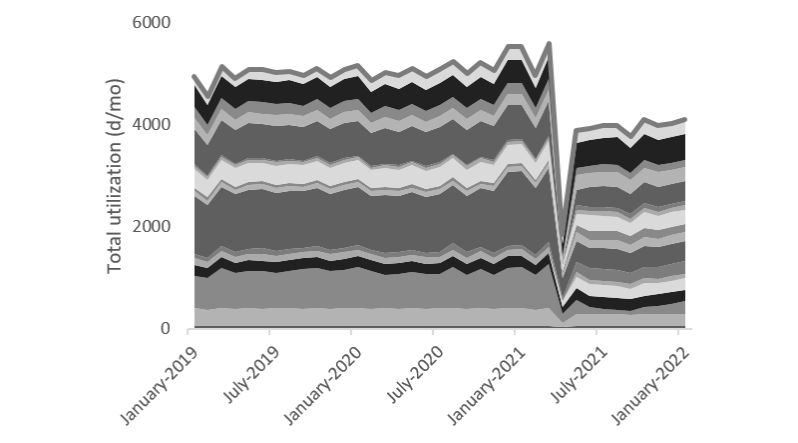
Utilization days per month for wheelchairs. As of May 2021, wheelchairs were fully delivered on the basis of the outsourcing contract. Before this date, 90% were locally owned by care departments. Color tones relate to different care locations. Outcomes of linear regression model 1a: outsourcing: *P*=.03, portal: *P*=.82, month: *P*=.50.

**Figure 3:**
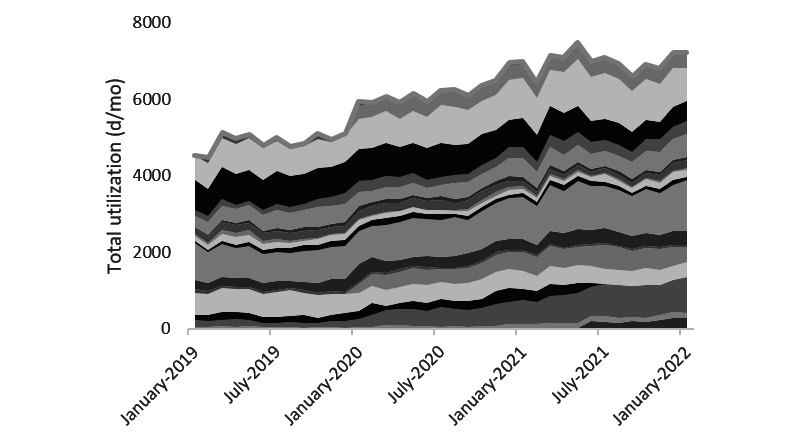
Utilization days per month for anti–pressure ulcer mattresses. Anti–pressure ulcer mattresses were delivered on an outsourcing basis during the entire period shown in the graph. Color tones relate to different care locations. Outcomes of linear regression model 1b: portal: *P*<.001, month: *P*<.001.

Figure 2 is based on our analysis of the utilization data and shows that wheelchair utilization decreased significantly (*P*=.03) by 1106 (SD 106) days per month on average (January 2019 to March 2021: n=5079, May 2021 to January 2022: n=3972). This reduction in total rental time amounts to the most clearly visible pattern across the 8 devices included in the intervention. For the other types of devices, such as lifting slings and low-low beds, we also observed considerable reductions in utilization days, albeit with a less consistent drop. The total number of utilization days of anti–pressure ulcer mattresses gradually increased over time (Figure 3). On the basis of on-site meetings, this increase can be explained by an increase in the number of patients with a high care burden. After the implementation of the web portal, this rising pattern stabilized, which was confirmed by the regression model (*P*<.001).

**Figure 4:**
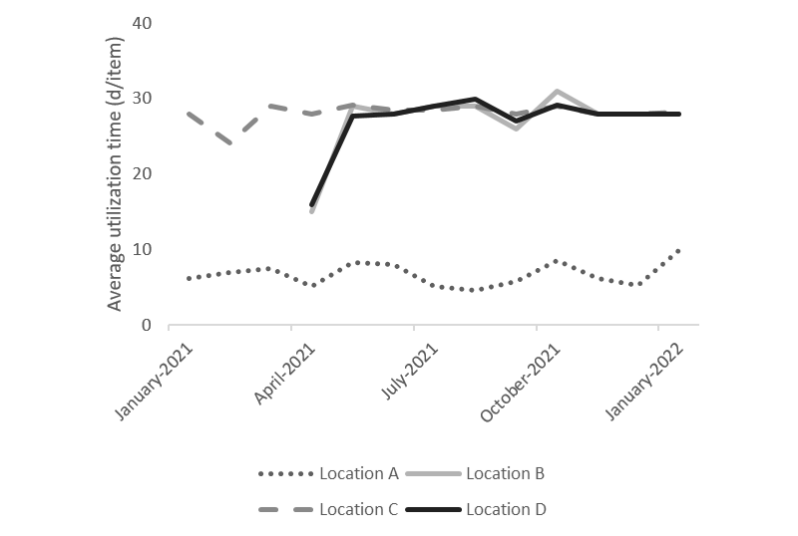
Average rental time per rented device per month of wheelchairs for 4 care locations. Outcomes of linear regression model 2a: outsourcing: *P*=.11, portal: *P*=.91, month: *P*=.95, location: *P*<.001.

**Figure 5:**
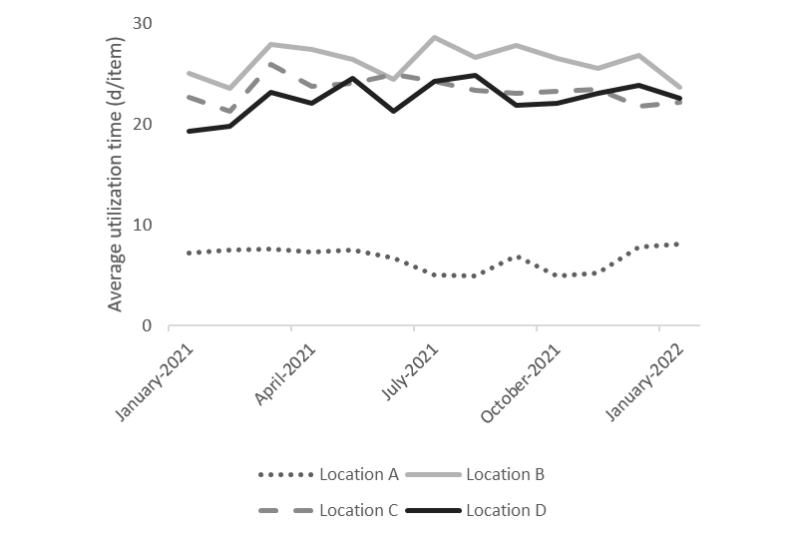
Average rental time per rented device per month of anti–pressure ulcer mattresses for 4 care locations. Outcomes of linear regression model 2b: portal: *P*=.71, month: *P*=.73, location: *P*<.001.

As indicated in Figures 4 and 5, the average rental time per rented device per month did not change over time for either wheelchairs or anti–pressure ulcer mattresses after the intervention. We observed similar patterns for the other types of devices.

#### Costs of Renting Medical Equipment

The cost of renting anti–pressure ulcer mattresses was on average US $32,381 per month after the intervention compared with US $25,445 per month before the intervention. The cost of renting wheelchairs was on average US $4501 per month after the intervention. No accurate data were available on ownership costs for wheelchairs, including the actual purchase price and the maintenance, procurement, storage, and material-handling costs. In addition, for both types of devices, there were no data available on the historical patterns of care delivery volumes and the disease burden of patients. Hence, a valid comparison of costs before and after the intervention was deemed infeasible by the researchers involved as well as the managers at the care provider under study.

#### Ordering and Delivery Time

The questionnaire results show how nurses perceived the ordering and delivery times before and after the intervention. Nurses who ordered both types of devices via the web portal reported statistically significant reduced ordering times for both wheelchairs (*P*=.04) and anti–pressure ulcer mattresses (*P*=.03) and statistically significant reduced delivery times for wheelchairs only (*P*=.01). Nurses who only ordered anti–pressure ulcer mattresses via the web portal and ordered wheelchairs via other channels reported reduced ordering times, albeit not statistically significant, for both devices (*P*=.35 and *P*=.26). These results are summarized in Table 5. Gauging the perceived changes in delivery time, nurses rated the item *devices are available faster* with a score of 2.2 (SD 1.1; using a 5-point Likert scale ranging from 1=*fully agree* to 5=*fully disagree*), indicating some improvement.

**Table 5:** Reported change in ordering (min) and delivery (d) times for wheelchairs and anti–pressure ulcer mattresses after implementation of the web portal^a^.

			Ordering time (min)									Delivery time (d)						
			Before, mean (SD)		After, mean (SD)		δ		*P* value		Before, mean (SD)			After, mean (SD)		δ		*P* value
**Ordering via the web portal (both devices; n=28)**																		
	Wheelchair	9.9 (7.4)		7.2 (3.1)		−2.7		.04		3.8 (2.1)			3.3 (1.8)		−0.5		.01	
	Mattress	10.7 (7.9)		7.6 (3.7)		−3.1		.03		2.4 (0.9)			2.1 (0.5)		−0.3		.07	
**Ordering mattress via the portal and wheelchair via email or telephone (n=10)**																		
	Wheelchair	6.8 (6.4)		5.5 (3.9)		−1.3		.35		4.4 (1.9)			4.6 (1.8)		0.2		.59	
	Mattress	8.2 (8.4)		6.0 (3.4)		−2.2		.26		2.6 (0.9)			2.5 (1.2)		−0.1		.34	

^a^*P* values are based on a paired 2-tailed *t* test between ordering time and delivery time before and after the intervention.

#### Quality of Care and Compliance With Safety and Hygiene Standards

Compliance with safety and hygiene standards was measured by responses to the statement *there are fewer uncertified devices at the location*. This item was rated positively by nurses who ordered both device types via the web portal with a score of 1.9 (SD 1.0; using a 5-point Likert scale ranging from 1=*fully agree* to 5=*fully disagree*). Of the 45 respondents, 22 (49%) fully agreed that there were fewer uncertified devices.

On the basis of the averaged questionnaire responses (Table 6), nurses did not report any difference in perceived patient satisfaction, care quality, safety, and care outcomes when comparing the old and new ordering processes for both device types.

**Table 6:** Assessment of the intervention with respect to the quality of care (rated using a 5-point Likert scale ranging from 1=fully agree to 5=fully disagree)^a^.

		Patients are more satisfied, mean (SD)	Care quality is higher, mean (SD)	Care is safer, mean (SD)	Better care outcomes, mean (SD)	Score, mean (SD)
Ordering via the web portal (n=28)		2.3 (1.0)	2.3 (1.2)	2.3 (1.2)	2.3 (1.2)	2.3 (1.1)
**Ordering mattress via the portal and wheelchair via email or telephone (n=10)**						
	Wheelchair	2.6 (1.0)	2.3 (0.9)	2.6 (0.9)	2.3 (0.9)	2.4 (0.9)
	Mattress	2.4 (0.9)	2.1 (0.8)	1.9 (0.8)	2.0 (0.8)	2.1 (0.8)
Ordering via a different method (n=7)		2.4 (1.4)	2.1 (1.1)	2.3 (1.3)	2.4 (1.5)	2.3 (1.3)
Average		2.4 (1.0)	2.2 (1.0)	2.3 (1.1)	2.3 (1.1)	2.3 (1.0)

^a^Effects of ordering via web portal versus effects of ordering mattress via web portal (wheelchair): *P*=.72, effects of ordering via web portal versus effects of ordering mattress via web portal (mattress): *P*=.53, effects of ordering via web portal versus effects of ordering via different method: *P*=.99, effects of ordering mattress via web portal (wheelchair) versus effects of ordering mattress via web portal (mattress): *P*=.39, effects of ordering mattress via web portal (wheelchair) versus effects of ordering via different method: *P*=.83, and effects of ordering mattress via web portal (mattress) versus effects of ordering via different method: *P*=.69 (average scores, based on a 2-tailed *t* test with unequal variances).

## Discussion

### Principal Findings

This study addresses the feasibility of a 2-fold logistical intervention aimed at outsourcing the management of reusable medical devices and the introduction of a web portal facilitating the ordering and use of these devices. The findings show that, with respect to acceptability, user satisfaction with the ordering and delivery process was high. Concerning preliminary efficacy, a reduction in the required number of wheelchairs, which was significantly related to outsourcing (*P*=.03), was observed. The increase in the required number of anti–pressure ulcer mattresses stabilized after the introduction of the web portal. For both wheelchairs and anti–pressure ulcer mattresses, we found shorter reported ordering and delivery times, but rental times per device did not reduce. Hence, the web portal may support the ordering and delivery processes, but there is no indication that it triggers a more efficient use of devices. After the intervention, a higher degree of device certification was reported, thereby increasing compliance with safety and hygiene standards. In theory, these improvements should translate into better outcomes in terms of costs and the quality of care. However, based on the findings, we could not establish that a reduction in overall costs had been achieved, and nurses did not report improvements in safety and the quality of care. Although, for all 8 types of reported medical devices, the implementation of the intervention was successful, based on observations and interviews, several challenges were highlighted related to improving the chain-wide management of medical devices. These challenges relate to the diverse nature of the supply base and complexities with having multiple care financers. In essence, these findings show the difficulties of managing, and catering to, the various stakeholder interests involved in the care chain, and this will be more elaborately reflected upon in the following subsection.

### Comparison With Previous Research

#### Improving Health Care Logistics Performance

The literature on health care logistics suggests that better management of medical devices—for example, through better forecasting, standardized replenishment, and delivery procedures—supported by materials management information systems can lead to a reduction in device stock and increase the occupancy rate of devices [2,7]. In terms of the outcomes of the care process, quality can be improved by reducing device stock-outs and delivery disruption. Hence, it is not surprising that, for health care managers, anticipated cost savings and quality improvements are important drivers when considering new ways of managing medical devices [27,28]. To a certain extent, our findings concur with the outcomes of these previous studies. The ordering process, device utilization, and compliance with safety and hygiene standards certainly improved after the outsourcing of reusable medical devices and the implementation of a web portal for ordering these devices. Indeed, the web portal is an important precondition in that it supports the ordering process and increases the measurability of device management. Nevertheless, we also observed that improvements related to reduced costs and the increased quality of care are not necessarily as straightforward as often assumed. We discuss potential reasons for this discrepancy in the next subsection.

#### Measurability of Costs and Quality of Care

We recognize that there is a lot of freedom in deciding which expenses and savings to include in comparing the outcomes of an intervention, and, as a result, establishing changes in overall costs is especially challenging [1]. Our case and the potential cost savings of the intervention provide a good example. The health care provider we studied did not account for the human resource costs related to the devices it owned (eg, costs related to maintenance and storage). Moreover, the provider did not take service delivery costs into account when evaluating the expenditure on devices. Similarly, an increase in expenditure on devices, as we observed for anti–pressure ulcer mattresses, may be misinterpreted as a rise in the costs of device management, rather than, at least in part in our case, an increase in patient volume and care burden. More generally, health care providers often have to deal with low-visibility and low-quality data [2,7]. Although providers increasingly work with resource planning systems (eg, ERP) and medical record information systems, and they are increasingly standardizing care service products, such as diagnosis-related treatment combinations, the transparency of costs in the health care sector remains an issue [29,30].

With respect to the quality of care, it would be highly beneficial if health care providers were able to show positive effects from better device management. Our questionnaire indicated that nursing staff did perceive an increase in the speed of device delivery and improvements in terms of compliance with safety and hygiene standards. We would expect this to contribute to the satisfaction of patients by reducing waiting time and increasing the available capacity, that is, the amount of time nurses have available for the direct care process. However, indicators related to patient satisfaction and safety remain hard to monitor because quality inspections are usually based on samples and periodic inspections. This exemplifies the usefulness of using patient-reported outcomes and experience measures [31,32], as well as linking such patient reports with clinical health records [33]. Such improved transparency should be considered not only for meeting external accountability demands but also for being able to demonstrate the effective rollout of improvements internally.

#### Structurally Embedding Organizational Changes

Our findings, supported by insights from previous studies [7,29-33], illustrate how transparent care processes and outcomes are key preconditions for structurally improving chain-wide care delivery and organizational changes. Because of various hidden costs and limited quality performance metrics, it was challenging for managers to show the true costs related to owning, as against renting, medical devices. Hence, it was difficult to justify the rental costs involved with outsourcing where all the costs (hidden or open) are part of the rental fee. At the same time, nursing staff noted that the intervention was rolled out on a relatively small scale in terms of the number of included types of equipment, leading to a confusing mixture of both new and old ordering procedures and complex coordination. This resulted in a situation where nursing staff felt that they were receiving insufficient top-down support, whereas, at the same time, top management perceived limited success from the intervention because not all nursing staff adhered to the new procedures. However, ultimately, it is likely that the studied intervention does lead to quality improvement and cost savings, yet it remains difficult to measure such improvements objectively.

Reflecting on the studied case enables us to provide recommendations for practice. Given the recently increased EU standards regarding the traceability and safety of medical devices [12], the presented intervention is of importance for medical device manufacturers, suppliers, and care providers. When looking at the dynamic circumstances of daily care delivery, one cannot expect manufacturers to achieve full compliance with standards by themselves. Hence, we recommend that medical device suppliers and care providers work jointly to improve medical device use in terms of efficiency, quality, and safety through outsourcing agreements supported with IT for care personnel. Moreover, by ensuring traceability and certification, these practices may also support the accessibility of innovative medical devices, another area that seems to be becoming more complex owing to the new EU standards [34,35].

Nevertheless, it seems that realizing a broad rollout of such an intervention and obtaining benefits in terms of cost savings and quality improvements remain a challenge, particularly for chain-wide care providers. This is especially the case because they are dealing with an extensive and complex device supply base. Top management needs to be aware of the related issues and challenges, particularly in a setting of care delivery that crosses the traditional boundaries of curative, long-term, and home care domains. For practice to structurally benefit from solutions such as outsourcing and web ordering portals, it is recommended to aim at reducing the complexity of the supply base, improving communication and support toward nursing staff, and establishing clear performance measures for evaluation purposes.

### Limitations

This study assessed the feasibility of an intervention based on qualitative and quantitative data collected before and after the intervention. Several limitations warrant mention. First, although our data indicate clear improvements over time, there are several potentially confounding factors that have not been considered explicitly; for example, although care registration data did not indicate considerable changes in patient numbers or disease burden, this was not part of the statistical analyses. Other examples include developments in treatment methods that may affect device use as well as disruptions in the supply and care process, such as those seen during the COVID-19 pandemic.

Second, our study took place against the backdrop of the introduction of the 2021 EU Medical Device Regulation. This context places our results within a framework of heightened standards for the traceability and safety of medical devices and shows a glimpse of the complex landscape that health care providers must navigate within the newly imposed regulatory constraints.

Third and last, the questionnaire on user experience that provided relevant insights that add to the basic device data was sent out only after the intervention, and it was targeted at nursing staff and not at patients. As such, we lack firsthand experiences from the latter group. This is especially relevant because our respondents did not report any clear effects on patient satisfaction or any effect on care quality, which provides an important direction for further study. This again stresses the importance of measuring patient experiences and outcomes over time for both care and research purposes.

### Conclusions

The integrated management of medical devices should lead to reduced costs and fewer required devices, higher quality of care, and reduced material waste. This feasibility study confirms this potential when it comes to acceptability, implementation success, and the preliminary efficacy of the presented intervention. Nevertheless, at a time when the integration of care chains is becoming more important, our research also highlights some of the difficulties when putting integration into practice; for example, in a health care system context that is based on market principles, it remains an immense challenge to achieve a more coherent way of managing medical devices, even for a single care provider when operating across traditional health care boundaries. In addition, the ongoing challenges in achieving transparency on prices and on the quality of care again prove key to measuring the efficacy of integrated medical device management which, in turn, is pivotal to achieving long-term implementation.

## Appendix

Multimedia Appendix 1 Interview guide.

Multimedia Appendix 2 User experience and care quality questionnaire.

## Abbreviations

ERP: enterprise resource planning
EU: European Union

## Data Availability

The data sets generated and analyzed during this study are not publicly available owing to confidentiality issues and agreements made with the case sites but are available from the corresponding author on reasonable request.
