# Supplementary material for: Outsourcing the Management of Reusable Medical Devices in a Chain-Wide Care Setting: Mixed Methods Feasibility Study
Source: Interact J Med Res. 2023 Sep 19;12:e41409. doi: 10.2196/41409 (PMC10548324; doi:10.2196/41409)
Supplement: Multimedia Appendix 1 [file ijmr_v12i1e41409_app1.docx]

**Multimedia Appendix 1 – Interview Guide**

The following questions were translated from Dutch. The use of open ended questions allows the research for a natural follow up of interviewees' responses to discussed phenomena.

**Introduction**

Thank you for taking the time to participate in this survey. My name is <name>, and I am currently conducting research on the renting and purchasing of medical devices within <case site>. I am interested in understanding what is needed in practice regarding renting or purchasing, implementation of the online portal, and how the current situation compares to a desirable future situation. I would also like to know the implications of different options for different departments.

This interview will be recorded, transcribed and coded. Only the researchers involved in this project will have access to the data, which will be securely stored for a maximum of 5 years. You can withdraw from this study at any time, without giving a reason. Do you give consent to participate?

Yes

No

1. **Background**

a) What is your role within the organization?

b) Which category of medical devices?

1. **Main Question**

Current management of devices

a) To what extent are medical devices currently moving smoothly through the care chain? (Can you provide examples?)

b) Is there a distinction between intramural and extramural care, or is there complete integration?

c) What methods are known within the organization for organizing logistics?

d) Is it clear what medical devices are present on the department and who owns them?

e) Are stocks centrally maintained at certain locations within the organization?

f) Is there insight into the current stock/management of resources?

g) Who or which department is responsible for assigning/requesting medical devices?

h) How are fluctuations in demand currently addressed?

1. **Barriers for device management**

Where the aforementioned device management does not go smoothly,

a) How do you notice this?

b) What are the consequences of this? (for your work, the patient, the organization)

c) What are the current obstacles to smoothly moving medical devices? To what extent do the following aspects play a role:

- Availability (ordering, delivery time, level of insight into the availability and management of aids)
- Autonomy (freedom of choice for the professional and patient)
- Maintenance (by the organization or supplier)
- Financing (how do you deal with transitions between specialized care and long-term care insurance, among others)
- Purchase/rental/reuse of resources (with regard to hygiene/regulations)
- Communication, cost structures, legislation, unclear processes, etc.

1. **Transition to outsourcing**

a) What are currently considerations when it comes to the decision to rent or purchase?

b) How does the decision to outsource (rent) a larger number of medical devices affect the below aspects of medical device management?

- Costs: Why and in what way?
- Stability of demand
- Quality
- Safety
- Availability
- Speed

c) In case you have experience with the new rental process, what are current flaws in medical device management, or barriers for improving the rental process?

**5. Online portal**

a) What do you or your colleagues think of the online portal that will be / was implemented to facilitate the ordering of medical devices?

b) To what extent does the online portal help to eliminate the obstacles discussed earlier in this interview? (Or what are the expectations regarding this?)

c) What were barriers for implementing the online portal, or for improving how the portal was currently implemented?
