# Supplementary material for: Outsourcing the Management of Reusable Medical Devices in a Chain-Wide Care Setting: Mixed Methods Feasibility Study
Source: Interact J Med Res. 2023 Sep 19;12:e41409. doi: 10.2196/41409 (PMC10548324; doi:10.2196/41409)
Supplement: Multimedia Appendix 2 [file ijmr_v12i1e41409_app2.docx]

**Multimedia Appendix 2 - Questionnaire**

Description

Evaluation of the mobility aids rental and portal

Since 2021, many mobility aids such as wheelchairs are no longer stored and maintained at <case site>, and are exclusively requested and delivered by <logistical partner>. This rental agreement is supported by the new online portal, which replaces the previous ordering process.

In order to obtain a better understanding of the functioning of the new working method regarding the use of care aids, from the perspective of care providers and other <case site> employees, we kindly ask that you to complete this questionnaire.

This research is being conducted by researchers from the University of Groningen on behalf of <case site> and <logistical partner>.

Question

Data and consent

Your anonymity is guaranteed. The data collected in this study will only be used by the researchers and clients and will be treated in accordance with the General Data Protection Regulation (GDPR). Participation in this study is completely voluntary and you can stop with the survey at any time.

Do you confirm that you are 18 years or older, you understand the purpose of this research, you want to participate in this research, and that you give permission to use your answers for scientific research?

Yes

No

**General questions**

Please answer the general questions first.

(If you work at multiple sites or have multiple job functions or disciplines you contribute to, please fill in these questions form the perspective of the task that takes the most of your time.)

Question

What is your age?

Younger than 18

18 up until 29

30 up until 39

40 up until 49

50 up until 59

60 up until 69

Question

At which <case site> location do you work?

<case site location 1>

<case site location 2>

(…)

<case site location 19>

Question

In which discipline are you employed?

Somatic

Psychogeriatric

Revalidation and recovery

Living - care center

Other (e.g. crisis, ELV, hospice care, Korsakov)

Different from listed, namely….

Question

What is your job function?

Care assistant

Nurse

Care coordinator

Team lead

Location lead

Ergo therapist

Physician

Different from listed, namely…

Question

How long have you been working in this job?

One till six months

Six to twelve months

One year or longer

**Content questions**

Please answer the following series of questions on the ordering process of the care devices.

Question

I order these devices at <logistical partner>

Via the online portal

Via a different channel (phone or e-mail)

I do not order devices

Follow up question when respondent uses a different channel:

Why do you not use the online portal to order the devices?

<fill in answer>

Follow up question when respondent does not order devices:

Why do you not order the devices?

<fill in answer>

Follow up question when respondent uses another channel or does not order devices:

Did you order devices via the online portal in the past?

Yes

No

Follow up question when respondent used the portal in the past:

Why did you stop ordering devices via the online portal?

<fill in answer>

Follow up question when respondent is using the portal for ordering devices or has used the portal in the past:

Please answer the following series of questions on the ordering process of devices via the online portal.

Question (if respondent indicated to use the portal)

How many minutes do you spend on ordering of….

A wheelchair

<fill in response>

An AD matrass
<fill in response>

Question (if respondent indicated to have used the portal in the past)

How many minutes do you spend on ordering (via the portal) of….

A wheelchair

<fill in response>

An AD matrass
<fill in response>

Question

After ordering a wheelchair, what is the average delivery time?

The same work day

The next work day

Within two working days

Within three working days

Within four working days

Within five working days

Longer than a work week

Question

After ordering an AD mattress, what is the average delivery time?

The same work day

The next work day

Within two working days

Within three working days

Within four working days

Within five working days

Longer than a work week

Question

Before working with the online portal, how much time did you spend on the ordering of…

A wheelchair

<fill in answer>

An AD mattress

<fill in answer>

Question

Before working with the online portal, what was the average delivery time of a wheelchair?

The same work day

The next work day

Within two working days

Within three working days

Within four working days

Within five working days

Longer than a work week

Question

Before working with the online portal, what was the average delivery time of an AD mattress?

The same work day

The next work day

Within two working days

Within three working days

Within four working days

Within five working days

Longer than a work week

Question

Ordering via the online portal is…

|  | Completely agree | Somewhat agree | Neither agree nor disagree | Somewhat disagree | Completely disagree |
| --- | --- | --- | --- | --- | --- |
| Easy |  |  |  |  |  |
| Clear |  |  |  |  |  |
| Fast |  |  |  |  |  |

Question

The portal is…

|  | Completely agree | Somewhat agree | Neither agree nor disagree | Somewhat disagree | Completely disagree |
| --- | --- | --- | --- | --- | --- |
| Accessible |  |  |  |  |  |
| Stable |  |  |  |  |  |

Please answer the next series of questions on ordering medical devices via phone or e-mail.

Question

How many minutes do you spend on ordering via phone or e-mail of….

A wheelchair

<fill in response>

An AD matrass
<fill in response>

Question

What is the average delivery time for a wheelchair ordered via phone or e-mail?

The same work day

The next work day

Within two working days

Within three working days

Within four working days

Within five working days

Longer than a work week

Question

What is the average delivery time for an AD mattress ordered via phone or e-mail?

The same work day

The next work day

Within two working days

Within three working days

Within four working days

Within five working days

Longer than a work week

Question (only for respondents that ordered via portal)

Please fill in the table below on the ordering of medical devices.

|  | Completely agree | Somewhat agree | Neither agree nor disagree | Somewhat disagree | Completely disagree |
| --- | --- | --- | --- | --- | --- |
| Devices are available sooner |  |  |  |  |  |
| The choice of devices is larger |  |  |  |  |  |
| Care clients are more satisfied |  |  |  |  |  |
| It provides a better quality of care |  |  |  |  |  |
| Care is safer |  |  |  |  |  |
| The care result is better |  |  |  |  |  |
| There is a better understanding of the available devices in a department |  |  |  |  |  |
| There is a smaller amount of non-tested devices |  |  |  |  |  |
| Care clients can more easily transfer devices between locations/departments |  |  |  |  |  |

Question (for respondents that used phone or e-mail to order)

Please fill in the table below on the ordering of medical devices since the devices are available via rental.

|  | Completely agree | Somewhat agree | Neither agree nor disagree | Somewhat disagree | Completely disagree |
| --- | --- | --- | --- | --- | --- |
| Devices are available sooner |  |  |  |  |  |
| The choice of devices is larger |  |  |  |  |  |
| Care clients are more satisfied |  |  |  |  |  |
| It provides a better quality of care |  |  |  |  |  |
| Care is safer |  |  |  |  |  |
| The care result is better |  |  |  |  |  |
| There is a better understanding of the available devices in a department |  |  |  |  |  |
| There is a smaller amount of non-tested devices |  |  |  |  |  |
| Care clients can more easily transfer devices between locations/departments |  |  |  |  |  |
